# Supplementary material for: Unique Growth Pattern Presentation of a Papillary Renal Cell Carcinoma
Source: Diagnostics (Basel). 2022 Aug 6;12(8):1904. doi: 10.3390/diagnostics12081904 (PMC9406930; doi:10.3390/diagnostics12081904)
Supplement: Supplementary file 1 [file diagnostics-12-01904-s001.zip › diagnostics-1823504-supplementary.pdf]

Table S1. All genomic alterations detected in the histologically normal tissue.

Gene	Amino Acid Change	Coding	Variant ID	Locus	Allele Frequency	Transcript	Variant Effect
FGFR3	p.(T651=)	c.1953G>A	.	chr4:1807894	99.85%	NM_000142.4	synonymous
FGFR3	p.(L706=)	c.2118G>A	.	chr4:1808360	4.80%	NM_000142.4	synonymous
PDGFRA	p.(P567=)	c.1701A>G	.	chr4:55141055	88.20%	NM_006206.6	synonymous
PDGFRA	p.([S566=;P567=])	c.1698_1701delCCCAinsTCCG	.	chr4:55141052	11.80%	NM_006206.6	synonymous, synonymous
KIT	p.(L644P)	c.1931T>C	.	chr4:55594228	25.96%	NM_000222.3	missense
KIT	p.(R683Tfs*43)	c.2047_2048insCC	.	chr4:55595557	93.33%	NM_000222.3	frameshift Insertion
KIT	p.(R684G)	c.2050A>G	.	chr4:55595560	93.33%	NM_000222.3	missense
KDR	p.(?)	c.3849-2A>G	.	chr4:55946332	26.70%	NM_002253.3	unknown
APC	p.(T1493=)	c.4479G>A	.	chr5:112175770	100.00%	NM_000038.6	synonymous
EGFR	p.(G575R)	c.1723G>A	.	chr7:55232973	42.57%	NM_005228.5	missense
EGFR	p.(N808=)	c.2424T>C	.	chr7:55249126	3.05%	NM_005228.5	synonymous
MET	p.(T1011A)	c.3031A>G	.	chr7:116411992	3.83%	NM_001127500.3	missense
SMO	p.(A643Dfs*134)	c.1927_1928insAC	.	chr7:128851602	57.14%	NM_005631.5	frameshift Insertion
SMO	p.(?)	c.1936+1G>C	.	chr7:128851612	80.00%	NM_005631.5	unknown
BRAF	p.(T470K)	c.1409C>A	.	chr7:140481399	100.00%	NM_004333.6	missense
FGFR1	p.(D165=)	c.495T>C	.	chr8:38285916	33.33%	NM_001174067.1	synonymous
GNAQ	p.(D236G)	c.707A>G	.	chr9:80409407	3.20%	NM_002072.5	missense
RET	p.(L769=)	c.2307G>T	.	chr10:43613843	73.33%	NM_020975.6	synonymous
RET	p.(P785L)	c.2354C>T	.	chr10:43613890	4.31%	NM_020975.6	missense

PTEN	p.(P246=)	c.738G>A	.	chr10:89717713	15.49%	NM_000314.8	synonymous
FGFR2	p.(E368G)	c.1103A>G	.	chr10:123274815	6.94%	NM_000141.5	missense
HRAS	p.(H27=)	c.81T>C	COSM249860	chr11:534242	99.56%	NM_001130442.2	synonymous
ATM	p.(S333F)	c.998C>T	.	chr11:108117787	25.49%	NM_000051.3	missense
ATM	p.(I1688V)	c.5062A>G	.	chr11:108170497	4.50%	NM_000051.3	missense
ATM	p.(E2711Q)	c.8131G>C	.	chr11:108205816	85.71%	NM_000051.3	missense
PTPN11	p.(I514=)	c.1542C>T	.	chr12:112926922	3.30%	NM_002834.5	synonymous
CDH1	p.(T66A)	c.196A>G	.	chr16:68835605	8.70%	NM_004360.5	missense
TP53	p.(R282=)	c.846G>A	COSM44724	chr17:7577092	1.35%	NM_000546.5	synonymous
TP53	p.(P72R)	c.215C>G	.	chr17:7579472	47.55%	NM_000546.5	missense
SMAD4	p.(G145E)	c.434G>A	.	chr18:48575674	95.56%	NM_005359.6	missense
SMAD4	p.(L146R)	c.436_437delTTinsAG	.	chr18:48575676	95.65%	NM_005359.6	missense
SMAD4	p.(L146G)	c.436_437delTTinsGG	.	chr18:48575676	4.35%	NM_005359.6	missense
SMAD4	p.(V333A)	c.998T>C	.	chr18:48591835	11.65%	NM_005359.6	missense
SMAD4	p.(G384=)	c.1152C>A	.	chr18:48593401	47.37%	NM_005359.6	synonymous
STK11	p.(G276D)	c.827G>A	.	chr19:1221304	5.01%	NM_000455.5	missense

Table S2. All genomic alterations detected in the tumor tissue.

Gene	Amino Acid Change	Coding	Variant ID	Locus	Allele Frequency	Transcript	Variant Effect
PDGFRA	p.(R554G)	c.1660A>G	.	chr4:55141014	5.76%	NM_006206.6	missense
PDGFRA	p.([S566N;P567=])	c.1697_1701delGCCC AinsACCCG	.	chr4:55141051	7.01%	NM_006206.6	missense, synonymous
PDGFRA	p.(P567=)	c.1701A>G	.	chr4:55141055	92.99%	NM_006206.6	synonymous
PDGFRA	p.(R822G)	c.2464C>G	.	chr4:55152032	100.00%	NM_006206.6	missense
KIT	p.(?)	c.1991-2_1991-1delinsC.A		chr4:55595499	3.57%	NM_000222.3	unknown
KIT	p.(?)	c.1991-2_1991delinsCCC		chr4:55595499	96.43%	NM_000222.3	unknown
KIT	p.(R683Tfs*43)	c.2047_2048insCC	.	chr4:55595557	100.00%	NM_000222.3	frameshift Insertion
KIT	p.(R684G)	c.2050A>G	.	chr4:55595560	100.00%	NM_000222.3	missense
KDR	p.(P1345=)	c.4035T>C	.	chr4:55946144	3.30%	NM_002253.3	synonymous
KDR	p.(P479=)	c.1437A>T	.	chr4:55972953	25.00%	NM_002253.3	synonymous
KDR	p.(P479=)	c.1437A>C	.	chr4:55972953	75.00%	NM_002253.3	synonymous
KDR	p.(P479A)	c.1435C>G	.	chr4:55972955	28.57%	NM_002253.3	missense
KDR	p.(P479T)	c.1435C>A	.	chr4:55972955	71.43%	NM_002253.3	missense
KDR	p.(N478I)	c.1433A>T	.	chr4:55972957	100.00%	NM_002253.3	missense
FBXW7	p.(L594V)	c.1780C>G	.	chr4:153245411	66.67%	NM_033632.3	missense
FBXW7	p.(S462P)	c.1384T>C	.	chr4:153249394	4.31%	NM_033632.3	missense
APC	p.(L1342S)	c.4025T>C	.	chr5:112175316	6.25%	NM_000038.6	missense
APC	p.(L1342S)	c.4025_4026delTAins CG	.	chr5:112175316	93.75%	NM_000038.6	missense
APC	p.(L1488=)	c.4464A>G	.	chr5:112175755	100.00%	NM_000038.6	synonymous
APC	p.(A1492=)	c.4476C>T	.	chr5:112175767	99.75%	NM_000038.6	synonymous

APC	p.(T1493=)	c.4479G>A	.	chr5:112175770	100.00%	NM_000038.6	synonymous
APC	p.(P1497=)	c.4491A>G	.	chr5:112175782	100.00%	NM_000038.6	synonymous
APC	p.(S1504=)	c.4512C>T	.	chr5:112175803	100.00%	NM_000038.6	synonymous
APC	p.(A1508=)	c.4524T>C	.	chr5:112175815	99.90%	NM_000038.6	synonymous
APC	p.(I1516=)	c.4548A>T	.	chr5:112175839	100.00%	NM_000038.6	synonymous
APC	p.(A1582P)	c.4744G>C	.	chr5:112176035	98.84%	NM_000038.6	missense
MET	p.(I816R)	c.2447T>G	.	chr7:116403132	100.00%	NM_001127500.3	missense
MET	p.(H855Qfs*3)	c.2564_2565insGCTG	.	chr7:116403249	100.00%	NM_001127500.3	frameshift Insertion
MET	p.(M1247I)	c.3741G>C	.	chr7:116423412	100.00%	NM_001127500.3	missense
SMO	p.(C217=)	c.651C>T	.	chr7:128845157	3.85%	NM_005631.5	synonymous
SMO	p.(Q228*)	c.682C>T	.	chr7:128845188	99.85%	NM_005631.5	nonsense
SMO	p.(T644S)	c.1931C>G	.	chr7:128851606	100.00%	NM_005631.5	missense
SMO	p.(P645del)	c.1933_1935delCCA	.	chr7:128851607	100.00%	NM_005631.5	nonframeshift Deletion
SMO	p.(?)	c.1936+1G>C	.	chr7:128851612	100.00%	NM_005631.5	unknown
ABL1	p.(W235*)	c.705G>A	.	chr9:133738305	52.30%	NM_005157.6	nonsense
RET	p.(L769=)	c.2307G>T	.	chr10:43613843	100.00%	NM_020975.6	synonymous
PTEN	p.(V133A)	c.398T>C	.	chr10:89692914	100.00%	NM_000314.8	missense
ATM	p.([D408E;L409N])	c.1224_1227delTCTTinsGAAC	.	chr11:108119818	13.51%	NM_000051.3	missense, missense
ATM	p.(L409A)	c.1225_1227delCTTinsGCA	.	chr11:108119819	78.38%	NM_000051.3	missense
ATM	p.(P862S)	c.2584C>T	.	chr11:108138015	100.00%	NM_000051.3	missense
ATM	p.(S864=)	c.2592T>C	.	chr11:108138023	33.33%	NM_000051.3	synonymous
ATM	p.(V3025L)	c.9073G>T	.	chr11:108236137	100.00%	NM_000051.3	missense

PTPN11	p.(I494V)	c.1480A>G	.	chr12:112926860	7.85%	NM_002834.5	missense
FLT3	p.(L610V)	c.1828T>G	.	chr13:28608228	9.80%	NM_004119.3	missense
FLT3	p.([N609=;L610=])	c.1827_1828delTTins CC	.	chr13:28608228	85.62%	NM_004119.3	synonymous, synonymous
FLT3	p.(N609T)	c.1826A>C	.	chr13:28608230	5.04%	NM_004119.3	missense
FLT3	p.([E608D;N609Y])	c.1824_1825delAAins CT	.	chr13:28608231	94.24%	NM_004119.3	missense, missense
FLT3	p.(E608V)	c.1823A>T	.	chr13:28608233	10.39%	NM_004119.3	missense
FLT3	p.(R607G)	c.1819A>G	.	chr13:28608237	100.00%	NM_004119.3	missense
FLT3	p.(F605C)	c.1814T>G	.	chr13:28608242	100.00%	NM_004119.3	missense
FLT3	p.(R437K)	c.1310G>A	.	chr13:28610180	85.71%	NM_004119.3	missense
FLT3	p.(?)	c.1310-2_1310-1delinsG.T		chr13:28610181	60.00%	NM_004119.3	unknown
FLT3	p.(?)	c.1310-2A>G	.	chr13:28610182	40.00%	NM_004119.3	unknown
RB1	p.(E554G)	c.1661A>G	.	chr13:48955545	10.95%	NM_000321.2	missense
RB1	p.([Q689H;N690Y])	c.2067_2068delGAins TT	.	chr13:49033930	93.10%	NM_000321.2	missense, missense
RB1	p.([Q689R;N690Y])	c.2066_2068delAGAin sGTT	.	chr13:49033929	5.17%	NM_000321.2	missense, missense
AKT1	p.(P42S)	c.124C>T	.	chr14:105246476	3.30%	NM_001014431.2	missense
CDH1	p.(N93S)	c.278A>G	.	chr16:68835687	14.83%	NM_004360.5	missense
TP53	p.(S20P)	c.58T>C	.	chr17:7579855	9.89%	NM_000546.5	missense
TP53	p.(F19C)	c.56T>G	.	chr17:7579857	100.00%	NM_000546.5	missense
ERBB2	p.(V794M)	c.2380G>A	.	chr17:37881051	4.85%	NM_004448.3	missense
ERBB2	p.(D880Mfs*31)	c.2638delG	.	chr17:37881445	100.00%	NM_004448.3	frameshift Deletion
ERBB2	p.(D880E)	c.2640T>G	.	chr17:37881448	100.00%	NM_004448.3	missense

ERBB2	p.(G882C)	c.2644G>T	.	chr17:37881452	60.87%	NM_004448.3	missense
SMAD4	p.(W99R)	c.295T>A	.	chr18:48575101	100.00%	NM_005359.6	missense
SMAD4	p.([I326=;A327S])	c.978_979delTGinsCT	.	chr18:48591815	100.00%	NM_005359.6	synonymous, missense
SMAD4	p.(Y328H)	c.982T>C	.	chr18:48591819	100.00%	NM_005359.6	missense
SMAD4	p.(H444L)	c.1331A>T	.	chr18:48603030	77.78%	NM_005359.6	missense
SMAD4	p.(R445=)	c.1335A>C	.	chr18:48603034	70.00%	NM_005359.6	synonymous
SMAD4	p.(R445=)	c.1335A>G	.	chr18:48603034	20.00%	NM_005359.6	synonymous
STK11	p.(E33K)	c.97G>A	.	chr19:1207009	3.15%	NM_000455.5	missense
